# Parental Self-Feeding Effects on Parental Care Levels and Time Allocation in Palestine Sunbirds

**DOI:** 10.1371/journal.pone.0113890

**Published:** 2014-12-04

**Authors:** Shai Markman

**Affiliations:** Department of Biology and Environment, University of Haifa - Oranim, Tivon, Israel; University of Missouri, United States of America

## Abstract

The trade-off between parents feeding themselves and their young is an important life history problem that can be considered in terms of optimal behavioral strategies. Recent studies on birds have tested how parents allocate the food between themselves and their young. Until now the effect of food consumption by parent birds on their food delivery to their young as well as other parental activities has rarely been studied. I have previously shown that parent Palestine sunbirds (*Nectarinia osea*) will consume nectar and liquidized arthropods from artificial feeders. However, they will only feed their young with whole arthropods. This provided a unique opportunity to experimentally manipulate the food eaten by parents independent of that fed to their offspring. Here, I hypothesized that parents invest in their current young according to the quality of food that they themselves consume. Breeding pairs with two or three nestlings were provided with feeders containing water (control), sucrose solution (0.75 mol) or liquidized mealworms mixed with sucrose solution (0.75 mol). As food quality in feeders increased (from water up to liquidized mealworms mixed with sucrose solution): 1) Parents (especially females) increased their food delivery of whole arthropod prey to their young. 2) Only males increased their nest guarding effort. Nestling food intake and growth rate increased with increasing food quality of parents and decreasing brood size. These results imply that increasing the nutrient content of foods consumed by parent sunbirds allow them to increase the rate at which other foods are delivered to their young and to increase the time spent on other parental care activities.

## Introduction

In optimal foraging theory, energy and time are considered as the main currencies involved in decision-making by foraging animals [1]–[3]. In studies of central-place foraging, where parents consume and bring food to their young at the nest, researchers have estimated energetic costs and benefits to the parent and the young [4], [5]. Such studies have usually ignored the benefits and costs associated with consuming and delivering types of food containing different nutrients, which should be taken into account when dealing with foraging behavior in general. Previous studies have usually dealt with the nature of food delivery to the young [6] or have focused on one type of prey to learn how parents allocate food between themselves and their young [7].

In most bird species, parents consume the same types of food that they deliver to their nestlings [8]. However, many frugivorous and granivorous species supplement nestling diets with arthropod prey that contains essential nutrients such as proteins [9]. Further, several studies have shown that single-prey-loading parents selectively transport large prey to their nestlings and eat smaller prey themselves [10]–[12]. This may lead to different ranges of prey types being consumed by parents and nestlings, however, not often does it lead to exclusive food types for parents versus their young.

In most food supplementation experiments, the parents both ate and delivered the supplemented food. In some of these experiments, food supplementation resulted in an increase in parental care activities and reproductive success [13]. However, observed parental care levels cannot be explained by the amount of food available for feeding the young alone, an explanation must include factors affecting the ability of the parents to provide this care. One such factor may be the influence of the type of food and its nutritional content consumed by the parent on the parent’s rate of food delivery to its young. To the best of my knowledge, this has not been experimentally tested (but see our previous studies on Palestine sunbirds) [14], [15]. To do so, an experimental system is required in which there is not only separation between the types of food consumed by the parents and their young, but also where the parents eat at least two foods that differ from one another in their nutritional content.

Nectarivorous birds, such as sunbirds (*Nectarinidae*) in the Old World, hummingbirds (*Trochilidae*) in the New World and honeyeaters (*Meliphagidae*) in Australia, fulfill their energy requirements mainly with concentrated sugar in nectar [16]. Nectarivorous birds complement the nectar with arthropods and pollen, which supply them with protein and other nutrients [17]–[20]. Outside of the period when nestlings are being reared, and when both floral nectar and arthropods seem to be abundant, Palestine sunbirds spend significantly more time foraging for nectar than for arthropods (on average 81% and 19% out of their total foraging time accordingly) [19], as also found for other nectarivorous bird species [21]–[23]. The time spent by sunbirds foraging for arthropods and nectar does not necessarily correlate with the amounts of these foods consumed by the birds [19].

As parent Palestine sunbirds do not feed nectar to their nestlings, but only arthropods [24], I assumed that parents would not feed their young an arthropod-containing solution. The rationale behind this is that parent Palestine sunbirds apparently feed their young only whole arthropods. In tests similar to the ones done with nectar alone, I found that parents readily feed on liquidized mealworms mixed with sucrose solution from a feeder, but did not feed it to their young [24]. That enabled me to supply the parents with nectar mixed with supplementary nutrients for feeding themselves, thereby to also manipulate their non-carbohydrate nutrient intake. This, therefore, allowed me to address the question: how parent sunbirds adjust their allocation of time and food between themselves and their young according to the different types of food that the parents themselves consume, and how does this consequently affect their reproductive output?

In light of the above, I hypothesized that the increasing quality of food consumed by the parents would affect their parental care levels and time allocation to different activities. I predicted that parents fed on a mixture of liquidized arthropods and sucrose solution, will perform higher levels of parental care than parents fed only on a sucrose solution, assuming that the arthropod-sucrose solution to be a more complete supplement than sucrose alone. In turn, parents fed only on a sucrose solution will demonstrate higher levels of parental care than parents offered only water. The rationale behind this prediction is that as the supplementary food becomes increasingly nutritious it will further increase their energy and nutrient intake and reduce the time spent by parents on finding these food items. This extra energy and/or time will allow parents to invest more effort in caring for their young.

To test these predictions, I supplemented the parents' diet with nectar and arthropods, and made observations at the nest and its associated feeder. I also used time-budget data, collected by a second observer, for the parents while they were away from the nest. This was done in order to assess the effect of each of the dietary supplements on the allocation of time by the parents among different activities. I quantified the effects of parental behavior upon nestling growth by making daily measurements of mass, tarsus length and bill length. This was done to assess the effect of parental feeding on nestling growth as a function of the parents’ own diet.

## Methods

### Study Species

Palestine sunbirds (previous common name: orange-tufted sunbirds) are small passerines (6–7 gr) that rear their young in hanging nests of woven grass, leaves, hair and spider webs [25]. In Israel, it is a common breeder in natural habitats from open woody areas to dry wadis in the desert, but also in areas of human habitation, where introduced flowering plants make nectar readily available. Further, this species does not seem to be affected by human presence and is relatively tame, for example, it will usually continue foraging even when an observer is 10 meters away from it. It is territorial, monogamous and multiple-brooded in any one season [26].

I studied this species in the Sede Boqer area in the Central Negev Desert (31°02′ N, 34°46′ E, 475 m above sea level), Israel. The study was conducted outside nature reserve areas. Birds were caught by using a trap cage (approved by the Israel Nature and Parks Authority permit number 2010/37548), then they were color ringed, measured and were immediately released. The experiment complied with the current animal protection laws of Israel. This study obeyed and followed welfare regulations of the Israeli “Committee to Evaluate Animal Subject Research” under The University of Haifa’s Animal Experimentation Ethics Committee permit 228/11.

### Experimental Procedure

All pairs included in the study were color ringed before the experiment was started. The pairs used in this experiment were feeding their first successful brood of the season. The nests that were included in the experiment contained natural brood sizes of two or three nestlings, with no hatching failures (i.e., the brood sizes equaled the clutch sizes).

I used artificial feeders to manipulate the parental food supply. One feeder was placed near each nest as a permanent food source. The feeder (150 ml) was hung ca. 5 m from each nest to enable simultaneous observation of both the nest and its associated feeder. I randomly assigned pairs of parents to one of three treatment groups in which one of three solutions in the feeders were made available to the birds. The three solutions were: 1) water alone (control); 2) sucrose solution (0.75 mol); or 3) mixed sucrose solution (0.75 mol) and liquidized mealworms (2.5 g mealworms in a 100 ml solution). The selected sucrose concentration (0.75 mol) is within the range of concentrations that Palestine sunbirds consume in nature [27]. The quantity of liquidized mealworms was chosen to be within the range of daily protein intake by Palestine sunbirds [28]. This fraction of protein in dry mass intake (1.6%) was found to maintain body mass of captive non-reproducing sunbirds captured in my study area [28]. Although mealworms contain nutrients other than protein, such as fat [29], there are no estimates of these requirements in Palestine sunbirds.

To measure the amount of solution that the birds consumed from the feeders, next to each feeder, I placed an inaccessible control feeder, and used it to calculate the effects of evaporation. Both accessible and inaccessible feeders were weighed (±0.01 gr) before and after each observation session. All the feeders used in this experiment were maintained full, and contained much larger amounts of food than could be consumed by the parent sunbirds in a day. The three treatments were used on broods of either 2 or 3 nestlings resulting in 6 treatment combinations of solutions and brood sizes.

I used a total of 37 pairs (i.e. 74 birds) during the breeding season which lasted from February to the end of July. This resulted in six pairs of parents in each of five treatment groups and one treatment group (parents rearing 3 nestlings and provided with water) with seven pairs. I provided the designated solutions to these pairs from about three days before the nestlings were expected to hatch until nestlings were 13 days old.

### Data Collection

One observer sat near the nest for an hour per day while using 7×50 binoculars. During this time, he recorded the amount of time the parents spent feeding at the feeder, parental activities such as parental feeding visit rate (i.e., visits to the nest while feeding the young), time spent at the nest (but not including time that was spent brooding), and time spent mobbing predators near the nest. Mobbing times were recorded as the total time (in seconds) that individuals approached and made their particular “mobbing call” at any potential predator that came within about 5 m of the nest. Some nest sites may have been more prone than others to the types of natural nest disturbances measured here, thereby eliciting a greater mobbing effort from those parents. However, these differences should be unbiased because of the random assignment of the experimental groups. This was confirmed by the lack of any significant difference in the time of disturbance caused by potential predators between the experimental feeder groups (F_2, 31_ = 0.07, p = 0.928) or between the brood sizes (F_1, 31_ = 0.75, p = 0.391), and in the lack of any interaction term between disturbance time in the experimental feeder groups and brood sizes (F_2,31_ = 0.17, p = 0.841).

At the same time, the second observer followed the color-banded parents and watched them while using 7×50 binoculars when the birds were away from the nest. The second observer watched each parent for half an hour during the one-hour daily observation sessions. This observer always watched the birds from at least 20 meters away, as not to disturb their normal activity. The first five minutes of each half-hour session were excluded from further analysis because the birds may have needed time to get used to the fact that an observer was following them. This also helped to avoid any bias due to location of the parents while they were performing more easily detected behaviors such as hovering in front of flowers. During the half-hour observations, the birds were sometimes out of view. Therefore, these periods were excluded from the data. Hence, the actual net observation times are termed later on as total observed activity time.

Every day, the first and the second observer changed roles between watching the nest and observing the parent while it was away from the nest. This was done to avoid bias in recording the birds’ activities due to observer effect. The first half-hour observation of every nest was started with the opposite parental sex from the previous day, to avoid any effect of a given parental sex on how its partner behaved during the subsequent half-hour session. The observer who followed the parents used a hand-held behavior event recorder (Psion Organizer II). The behaviors that were recorded were time spent: (1) foraging for floral nectar; (2) foraging for whole arthropods; (3) mobbing potential predators, away from the nest area; (4) displaying in front of females other than its mate (performed only by males); (5) chasing away intruder sunbirds; (6) sitting; and (7) flying. All data were collected daily between 0800 and 1400 hours for each pair of parents from nestling age 7 to 13 days.

The order of observations at different nests was rotated every day, balancing any time of day effects. The random and alternate assignment of pairs to treatment groups also meant that any differences between observation days, caused by changes in weather or season, would be likely to be balanced across experimental and brood size groups. This was confirmed by the lack of any significant difference in hatching dates, and therefore in the dates of data collection, between experimental feeder groups (F_2,31_ = 0.07, p = 0.928) or brood sizes (F_1,31_ = 0.16, p = 0.686), and in the lack of any interaction between hatch dates in experimental feeder groups and brood sizes (F_2,31_ = 0.09, p = 0.913).

All nestlings were weighed daily (usually between 14.00 and 16.00) to the nearest 0.01 g with an electronic field balance (Ohaus, Model GT200). The lengths of the tarsus and bill were measured to the nearest 0.1 mm using Vernier calipers. All data were collected daily for each nest from nestlings aged 7–13 days. Data were not collected after this, because nestlings older than 13 days usually do not gain mass and are also too mature to handle without causing premature fledging [14].

### Statistical Analysis

Data were reduced to single parameter estimates (i.e. means) for each parent or nest prior to analysis of variance (ANOVA). To estimate the effects of experimental feeder or brood size on parental care variables such as nest visit rate, the unit for analysis was the mean number of visits per hour for each bird. I also tested the effects of experimental feeder and brood size on time that parents spent feeding at the feeder, parental visits per nestling, time spent at the nest and time spent mobbing near the nest. The total parental visits and parental visits per nestling may not be independent, but are pertinent to different points of interest (for examples of this idea see [30], [31]). These data were then analyzed using ANOVA with respect to experimental feeder, brood size, and parental sex. ANOVA was also used to test the effects of experimental feeder and brood size groups on nestling growth variables at the end of the manipulation (when nestlings were 13 days old), but here each brood served as a unit of analysis.

The time budget data were analyzed using multivariate ANOVA (MANOVA), as usually done for time budgets [32]. Prior to analysis, all data were reduced to the proportion of the total net observation time spent by the parent in each activity, averaged for all the experimental period from nestling age 7 days old until 13 days old. These parameters were then arcsine-square root transformed to achieve normality [33]. As each activity may not be independent from other activities, I used a Pearson correlation matrix to assess whether the activities were significantly correlated with one another. Since none of the activities were significantly highly correlated with any other activity (i.e. all the significant r values were between r = −0.5 and r = 0.5), I included all the variables in the MANOVA model.

I further used the results of the univariate ANOVA tests for each parental activity, to test the sources of variation in the data. For the results of ANOVA’s where particular between-treatment group comparisons were of interest, I present the results of Tukey HSD pairwise comparison tests ([34] SYSTAT version 7, 1997).

## Results

### Feeder Use

As food quality in the feeder increased, the parents spent significantly more time feeding at the feeder and consumed more of the solution (Table 1). However, there were no significant differences between males and females nor between parents rearing different brood sizes in the time that was spent feeding, or the amounts consumed from the feeder (Table 1).

**Table 1:** The effects of the experimental feeder food solution (Food), brood size (Brood) and parental sex (Sex) in Palestine sunbirds on (a) the mean time that was spent feeding at a feeder per hour, and (b) the mean volume of food solution consumed per hour. The results of the ANOVA models are presented with *df*, MS, F- ratios, and P-values.

a) Time spent feeding				
Source	*df*	MS	F	P
Food	2	3710.71	24.14	<0.001
Brood	1	137.44	0.89	0.348
Sex	1	5.05	0.03	0.856
Food by Brood	2	44.3	0.28	0.75
Food by Sex	2	6.92	0.04	0.955
Brood by Sex	1	33.99	0.22	0.639
Food by Brood by Sex	2	11.34	0.07	0.928
Error	62	153.66		

b) Volume consumed				
Source	*df*	MS	F	P
Food	2	0.94	72.83	<0.001
Brood	1	0.04	3.26	0.075
Sex	1	0.01	0.81	0.369
Food by Brood	2	0.002	0.2	0.816
Food by Sex	2	0.022	1.76	0.179
Brood by Sex	1	0.027	2.09	0.152
Food by Brood by Sex	2	0.003	0.26	0.768
Error	62	0.012		

### Levels of Parental Care

**Figure 1:**
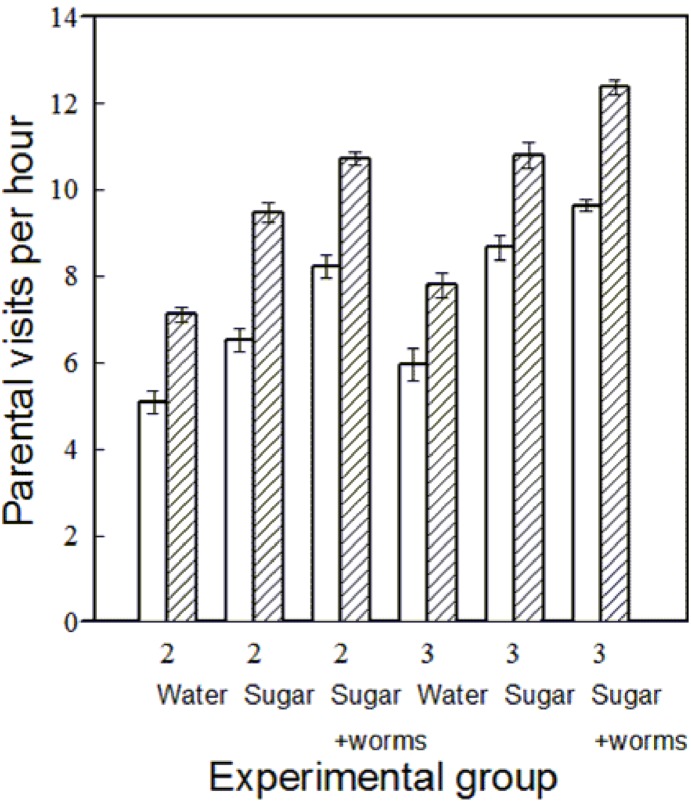
The number of parental visits per hour to the nest (means ± SE) by Palestine sunbirds plotted against brood size (2 or 3 nestlings), quality of food available in an artificial feeder, which was either water (Water), sucrose solution (Sugar) or sucrose and mealworm solution (Sugar and worms), and parental sex (blank: males; cross hatched: females). The values consist of the average for the whole seven days of the manipulation period (from nestlings being 7 days of age until 13 days of age) for each parent.

The food manipulation had a significant effect on parental visit rate (Table 2a). Both females and males increased their visit rates to the nest as food quality increased from water to sucrose solution, and to sucrose and mealworm solution (Table 2a, Fig. 1). There were significant effects of parental sex and of brood size on parental visit rates (Table 2a). Females had significantly higher visit rates than males, and parents visited larger broods more often (Table 2a, Fig. 1).

**Table 2:** ANOVA results for: (a) mean parental visit rates per hour; (b) mean time spent at the nest per parental visit; (c) mean time spent mobbing near the nest per hour, in Palestine sunbirds. The effects of food content in an artificial feeder (Food), brood size (Brood), and parental sex (Sex) are given. The results of the ANOVA models are presented with *df*, MS, F-ratios, and P-values.

a) Parental visit rates				
Source	*df*	MS	F	P
Food	2	90.15	223.59	<0.001
Brood	1	33.31	82.61	<0.001
Sex	1	102.49	254.2	<0.001
Food by Brood	2	1.6	3.99	0.023
Food by Sex	2	0.87	2.15	0.123
Brood by Sex	1	0.29	0.73	0.395
Food by Brood by Sex	2	0.45	1.12	0.331
Error	62	0.4		

b) Time spent at the nest				
Source	*df*	MS	F	P
Food	2	0.73	0.01	0.982
Brood	1	25.26	0.67	0.415
Sex	1	954.4	25.43	<0.001
Food by Brood	2	9.82	0.26	0.77
Food by Sex	2	228.93	6.1	0.003
Brood by Sex	1	0.01	0	0.984
Food by Brood by Sex	2	8.31	0.22	0.801
Error	62	37.52		

c) Time spent mobbing				
Source	*df*	MS	F	P
Food	2	5192.49	24.73	<0.001
Brood	1	5232.29	24.92	<0.001
Sex	1	126719	603.6	<0.001
Food by Brood	2	689.7	3.28	0.004
Food by Sex	2	3955.59	18.84	<0.001
Brood by Sex	1	1245.02	5.93	0.018
Food by Brood by Sex	2	118.09	0.56	0.573
Error	62			

**Figure 2:**
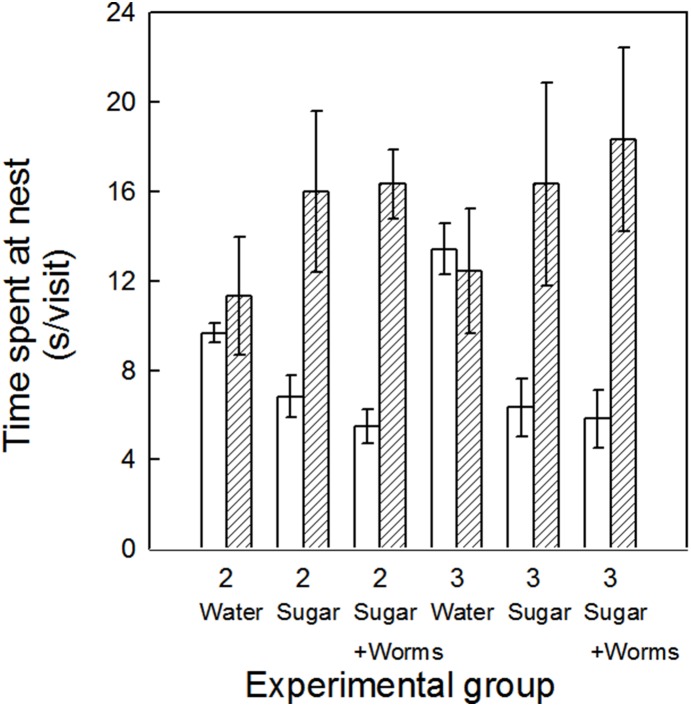
Time spent at the nest per parental visit (means ± SE) by Palestine sunbirds plotted against brood size (2 or 3 nestlings), quality of food available in an artificial feeder, which was either water (Water), sucrose solution (Sugar) or sucrose and mealworm solution (Sugar and Worms), and parental sex (blank: males; cross hatched: females). The values consist of the average for the whole seven days of the manipulation period (from nestlings being 7 days of age until 13 days of age) for each parent.

Females spent significantly more time at the nest than males (Table 2b, Fig. 2). Although the type of food in a feeder did not have a significant effect on the time spent at the nest (Table 2b, Fig. 2), there was a tendency of the females to spend more time at a nest with more food types in the feeder, with the opposite response in males. This resulted in a significant parental sex by food treatment interaction term (Table 2b).

**Figure 3:**
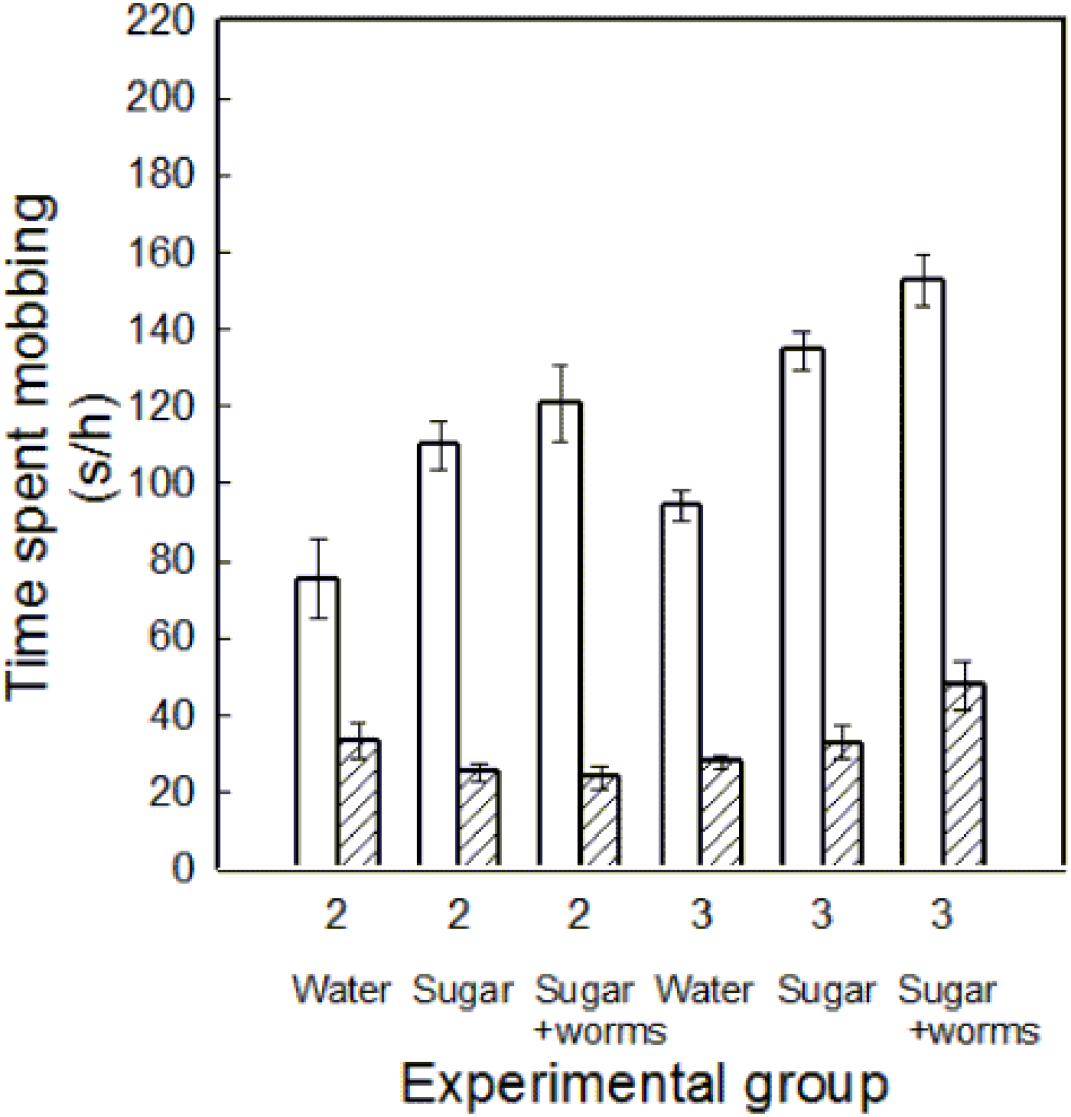
Time spent mobbing near the nest per hour (means ± SE) by Palestine sunbirds plotted against brood size (2 or 3 nestlings), quality of food available in an artificial feeder, which was either water (Water), sucrose solution (Sugar) or sucrose and mealworm solution (Sugar and worms), and parental sex (blank: males; cross hatched: females). The values consist of the average for the whole seven days of the manipulation period (from nestlings being 7 days of age until 13 days of age) for each parent.

Parental sex and food treatment both affected mobbing effort. The time that males spent mobbing was significantly greater than for females (Table 2c, Fig. 3). As predicted, males significantly increased their mobbing time with increasing nutritional quality of food offered (Fig. 3), while females did not (all Tukey HSD tests for females: p>0.05). As a result, there was a significant parental sex by food treatment interaction term (Table 2c). There was also a significant effect of brood size on mobbing time, with more mobbing effort for larger broods (Table 2c). However, as indicated by the significant parental sex by brood size interaction term, only the males, and not the females, increased their mobbing time when they reared larger broods (all Tukey HSD tests for females: p>0.05).

### Parental Visits Per Nestling

**Figure 4:**
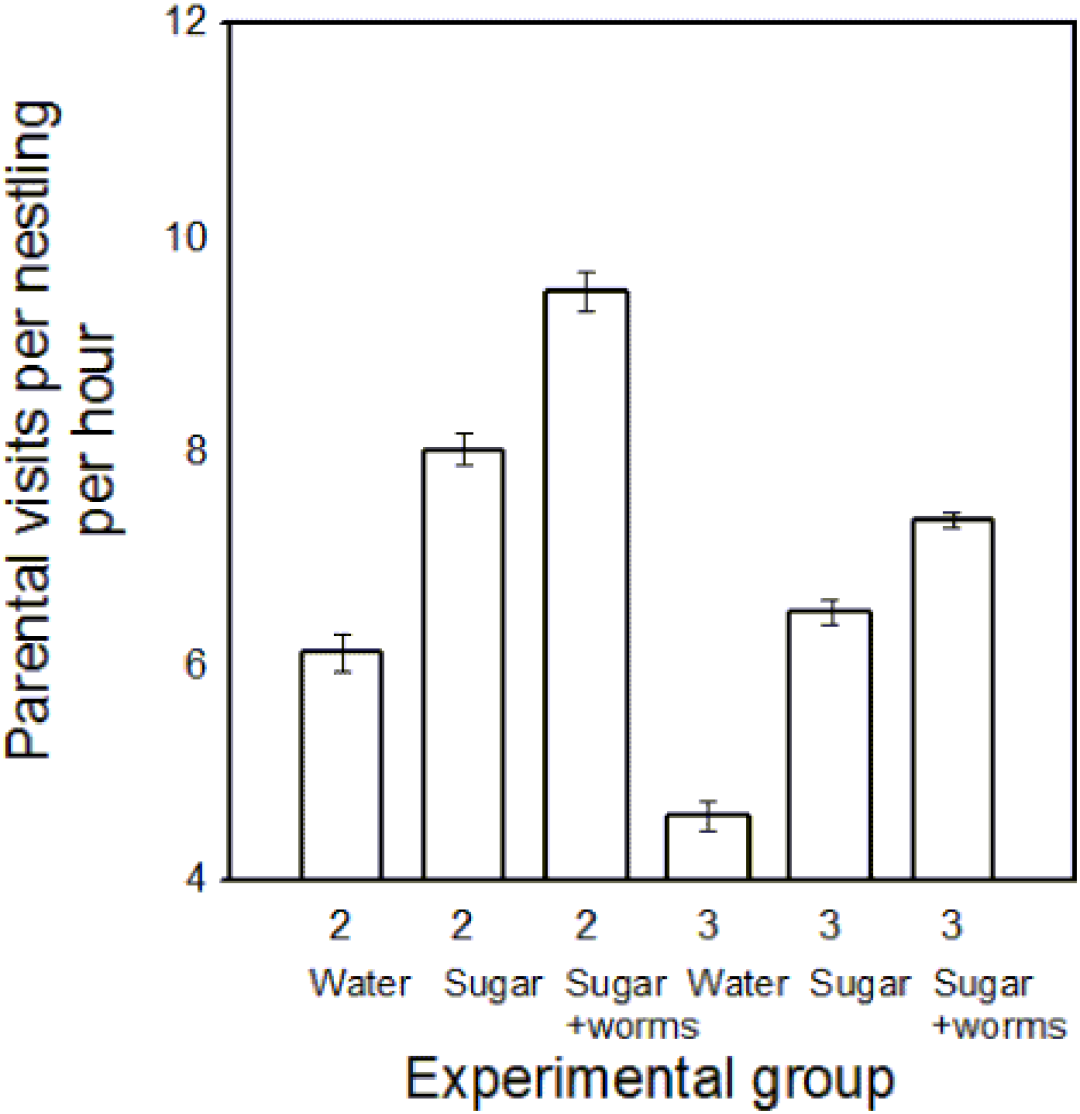
The number of parental visits per nestling per hour (means ± SE) by Palestine sunbirds plotted against brood size (2 or 3 nestlings), and quality of food available in an artificial feeder, which was either water (Water), sucrose solution (Sugar) or sucrose and mealworm solution (Sugar and worms). The values consist of the average for the whole seven days of the manipulation period (from nestlings being 7 days of age until 13 days of age) for each parent.

After testing for the number of parental visits and time spent at the nest during these feeding visits, I tested for differences in the number of parental visits per nestling and found that the number of feeding visits per nestling increased significantly with increasing food quality in the feeder and with decreasing brood size (all Tukey HSD tests p<0.01; Table 3a, Fig. 4). Although the total number of visits to the larger broods was higher than to the smaller broods, the number of visits per nestling was significantly lower in broods of three than in broods of two (all Tukey HSD tests p<0.001; Table 3a, Fig. 4). However, parents who reared three nestlings and were fed on sucrose solution had similar delivery rates of arthropods per nestling to parents who reared only two nestlings but were offered water in feeders (Tukey HSD test, p = 0.476). Furthermore, parents who reared three nestlings and were provided with sucrose and mealworm solution had higher delivery rates of arthropods per nestling than parents who reared only two nestlings, but were offered water in feeders (Tukey HSD test, p<0.001).

**Table 3:** ANOVA results for parental care levels and nestlings growth measurements in Palestine sunbirds: a) mean parental visits per nestling per hour; b) mean nestling mass, c) mean nestling bill length, d) mean nestling tarsus length at the end of the manipulation (day 13). The effects of food content in an artificial feeder (Food), and brood size (Brood) are given. Results of ANOVA models are presented with *df*, MS, F-ratios, and P-values.

a) Parental visits per nestling				
Source	*df*	MS	F	P
Food	2	29.90	229.13	<0.001
Brood	1	27.41	210.06	<0.001
Food by Brood	2	0.38	2.94	0.067
Error	31	0.13		

b) Nestling mass				
Source	*df*	MS	F	P
Food	2	1.36	5.29	0.010
Brood	1	3.79	14.73	<0.001
Food by Brood	2	0.04	0.15	0.856
Error	31	0.25		

c) Bill length				
Source	*df*	MS	F	P
Food	2	2.73	0.60	0.553
Brood	1	2.77	0.60	0.444
Food by Brood	2	0.21	0.04	0.954
Error	31	4.54		

d) Tarsus length				
Source	*df*	MS	F	P
Food	2	2.27	0.92	0.408
Brood	1	3.84	1.55	0.221
Food by Brood	2	0.69	0.28	0.756
Error	31	2.46		

### Nestling Body Mass and Body Size

**Figure 5:**
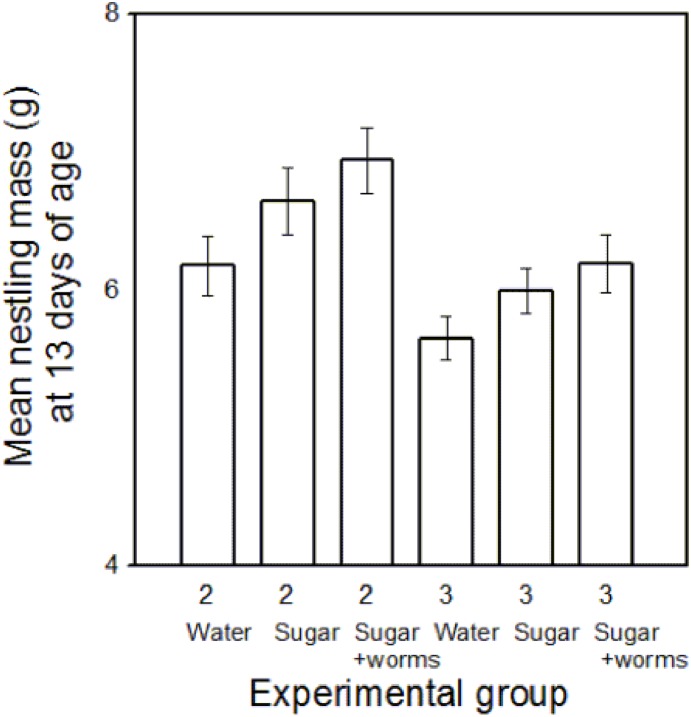
Palestine sunbird mean nestling mass at 13 days of age (means ± SE) plotted against brood size (2 or 3 nestlings), and quality of food available in an artificial feeder, which was either water (Water), sucrose solution (Sugar) or sucrose and mealworm solution (Sugar and worms).

Growth rate of nestlings was directly related to the level of care per nestling. The content of the food offered to the parents had a significant effect on mean mass of the nestlings attained at 13 days of age (the last day of the manipulation; Table 3b, Fig. 5).

Mean mass of a nestling at 13 days of age in broods of two was significantly higher than at that of broods of three (Table 3b, Fig. 5). Broods of three nestlings, whose parents fed on the sucrose and mealworm solution, received significantly higher number of parental visits per nestling than broods of two, whose parents were offered water. This presumably resulted in nestlings of both of these groups attaining similar maximum mass (Tukey HSD test, p = 1). Neither maximum bill nor tarsus lengths attained at age 13 days were affected by food content in the feeder or by brood size (Table 3c, 3d).

### Time Budgets

Parental sex had a significant overall effect (the multivariate effect) on the time spent by the parents at different activities (MANOVA: Wilks’ Lambda = 0.1815, F_8,55_ = 31.01, p<0.001). By further examining the differences within each activity, univariate ANOVA (see [35]) allows one to assess whether males differed from the females in the time spent at each activity. Male and female parents did not differ significantly from each other in the time spent foraging for floral nectar (F_1,62_ = 3.73, p = 0.057), flying (F_1,62_ = 0.11, p = 0.737), and sitting (F_1,62_ = 0.34, p = 0.560). However, females spent more time foraging for whole arthropods than males (F_1,62_ = 11.67, p = 0.001), while males spent significantly more time than females mobbing predators away from the nest (F_1,62_ = 42.37, p<0.001) and chasing intruder males (F_1,62_ = 16.00, p<0.001).

Brood size also had a significant overall effect on the time spent performing parental activities (Wilks’ Lambda = 0.5112; F_8,55_ = 6.57, p<0.001). The time spent sitting by the parents of broods of three was significantly lower than the time spent sitting by parents of broods of two (F_1,62_ = 19.97, p<0.001). The time spent by males displaying in front of other females also was greater for males rearing broods of two than broods of three (F_1,62_ = 5.92, p = 0.017). However, parents who reared three nestlings spent significantly more time foraging for floral nectar (F_1,62_ = 8.48, p = 0.005) and for whole arthropods (F_1,62_ = 4.45, p = 0.038) compared to parents rearing two nestlings. There were no significant differences between the parents rearing different brood sizes in the time they spent: mobbing predators away from the nest (F_1,62_ = 1.51, p = 0.222), and chasing intruders (F_1,62_ = 0.25, p = 0.617).

Food content in the feeder had a significant overall effect on the time spent on parental activities (Wilks’ Lambda = 0.2276; F_16,110_ = 7.53, p<0.001). The time spent on the following activities was significantly increased as the nutritional quality of food increased: foraging for whole arthropods (F_2,62_ = 3.76**,** p = 0.028), males displaying in front of other females (F_2,62_ = 15.47, p<0.001), and flying (F_2,62_ = 7.03, p = 0.001). The time spent foraging for floral nectar (F_2,62_ = 31.14, p<0.001) and sitting (F_2,62_ = 9.53, p<0.001) decreased with increasing food quality. The time spent mobbing away from the nest (F_2,62_ = 0.53, p = 0.589) and chasing intruder males (F_2,62_ = 0.76, p = 0.469) was not significantly affected by the food content in the feeder. The percent time spent foraging for nectar by the parents that were provided with sucrose or with sucrose and mealworm solution was significantly lower than those of the water treatment group (Tukey HSD tests p<0.05). However, there were no significant differences between the sucrose solution group and the sucrose and mealworm solution group in the time spent by parents foraging for nectar or for arthropods (all Tukey HSD tests p>0.05).

The parental sex by brood size interaction term in the MANOVA was significant (Wilks’ Lambda = 0.6295, F_8,55_ = 4.04, p<0.001). However, only the time spent sitting differed between males and females as brood size increased (F_1,62_ = 9.96, p = 0.002). This was because the males demonstrated greater differences in time spent sitting between broods of two and three than did females.

The brood size by food treatment interaction term in the MANOVA was not significant (Wilks’ Lambda = 0.907, F_16,110_ = 0.34, p = 0.991), neither was the triple interaction term of parental sex by brood size by food content (Wilks’ Lambda = 0.892, F_16,110_ = 0.400, p = 0.979), nor any of the univariate tests within these multivariate interaction terms (all p values>0.05).

## Discussion

In the present experiment, I offered parents either water, sucrose solution or liquidized mealworms in sucrose solution, in order to test whether they adjust their provisioning rates according to their own feeding rates on different food supplements. Thus, the effect of the manipulation was to create different levels of parental self-feeding in terms of energy and other nutrient intake and/or time spent self-feeding. By increasing the energy and other nutrient intake from the feeders, and/or by changing the time spent self-feeding, parents were apparently able to invest more in providing whole arthropods to their young.

In general, the increase in provisioning rate with the increase of food quality in the feeders that was available for parental self-feeding agrees with the predictions of the theoretical models of food-allocation decisions [7], [36]. Kacelnik and Cuthill [7] and Beauchamp et al. [36] predicted that parents would allocate the food they find between themselves and their young based upon maximizing their lifetime reproductive success. This means that parents should allocate food to their current young in a manner that will also allow them to maintain their own body condition and to survive for future reproductive attempts. In partial support of this idea, Antarctic petrel (*Thalassoica antarctica*) parents were shown to adjust the amount of food delivered to their current chick according to both the chick's needs and their own body condition, which will allow the parents to maximize their chances to survive for future breeding bouts [37]. Indeed, the present study confirms that parents that consume higher-quality food or feed themselves more efficiently should have more energy and time to invest in feeding their current nestlings. By contrast, provisioning models [4], [8], [38], [39] do not use the long-term calculation of lifetime reproductive success. Much more in line with the findings of the present study, these models predict that, as net energy gain from self-feeding in the parents increases, energy delivery to the nestlings will also increase. Here, I have additionally found that as the nutritional gain (and not only the energetic value) from self-feeding in the parents increased, energy and nutrient delivery to the nestlings also increased. Further, similar results were obtained from our previous work on this population showing that delivery rates to the young were affected by the energy content of the food consumed only by the parents [14], [15].

There were greater increases in the provisioning rates between the water and the sucrose solution-supplementation groups of parents compared to the increases between the sucrose and the sucrose and mealworm solution groups. This pattern suggests that the sucrose solution supplement had a stronger effect on the provisioning rate of arthropods, than the addition of liquidized mealworms to the parents’ diet. This difference in the effect of food supplements on the provisioning rate may be due to nectar being the major food source for parent sunbirds [19], [28]. Thus, sucrose solution apparently saved the birds more time, or further increased their energy gain than did the addition of liquidized mealworms to their diet.

As we found in our previous work [40], [41] and in the present experiment, sunbird nestlings were predominantly cared for by their mothers, at least in terms of provisioning activity and the time that parents spent at the nest. Although the food treatment did not have a significant effect on the time spent at the nest, females tended to stay longer at the nest when offered either sucrose or sucrose and mealworm solution than females that were offered only water. In contrast, males that received a sucrose or sucrose and mealworm solution spent less time at the nest than males that were offered water. This suggests that males and females differed in the way they increased their provisioning rate. While the males seemed to trade-off the number of their visits to the nest and the time they spent at the nest, females did not. This difference in the allocation of parental effort between the sexes may have been due to the males allocating more time to alternative activities. These alternative activities were mobbing away from the nest, chasing intruder males, and displaying in front of females other than their mates. This suggests that there are sex-specific patterns of time allocation to different activities in the Palestine sunbird [40], [41].

The males increased their mobbing time in proportion to more types of food in the feeders, while females did not. The increase in male mobbing effort is similar to the results of the experiment in which different sucrose concentrations were offered to the parents [14]. Increases in mobbing time probably resulted from different levels of extra time saved and/or extra energy gained that could have been dedicated to mobbing by the males who were feeding on different food types.

Males with larger broods mobbed for more time than those of smaller broods only near the nest. One reason for that might be that males with larger broods, which were feeding their young at a higher rate and therefore spent more time near the nest, were more prone to detecting potential predators. Another explanation might be that parents of larger broods were of a better quality and therefore were able to spend more time mobbing. This explanation would be further supported if mobbing was shown to be costly and therefore only parents of higher quality can afford to spend more time on mobbing. Since it was mostly the males that contributed more time to mobbing as brood size increased, it might be that the very low investment in mobbing by females is not a good indicator to assess their quality.

Parents who reared three nestlings and were provided with sucrose and mealworm solution had significantly higher delivery rates of arthropods than parents who reared only two nestlings but had been offered water in feeders. This resulted in similar mean nestling body masses in the two groups. Thus, the sucrose and liquidized mealworms apparently allowed an increase in parental effort, roughly equal to that needed to raise an extra nestling. The number of feeding visits per nestling, and therefore nestling mass at the end of the experiment, increased with more types of food in the feeder, reflecting the effect of parental self-feeding on nestlings’ growth. Males with larger broods spent less time than males with smaller broods displaying around other females. This may suggest that the level of parental care is traded-off against alternative mating opportunities [42].

The decrease in the time spent foraging for floral nectar by the parents who were provided with sucrose or sucrose and mealworm solution was associated with more time spent foraging for arthropods than in the parents that were provided with water only. This supports the idea that the time spent foraging for nectar is traded-off against the time spent foraging for arthropods. There were no significant differences in the time spent foraging for arthropods between the parents that fed on sucrose or sucrose and mealworm solution. However, the number of parental visits to the nest was higher for the parents that were fed sucrose and mealworm solution than in parents that were fed only sucrose solution. This may suggest that the parents whose food was supplemented with sucrose and mealworms were allocating more of the arthropods that they captured to feed their nestlings than parents that were supplemented with sucrose only. That might be possible since the parents that were feeding on the sucrose and mealworm solution received some of their own protein and other non-carbohydrates nutritional requirements, and therefore needed to consume fewer arthropods.

While parental self-feeding is an important factor that determines parental effort, as was demonstrated in the present study, there are other factors that may explain the observed parental care levels. Previous studies have demonstrated that parental investment is affected by the body condition of the parents and the nestlings and/or by food availability in time and space [43]–[49]. For example, it was shown that blood parasites and food availability acted together to affect parental effort in Tengmalm's Owls across different years [43]. However, other studies have demonstrated that food quality and quantity are not sufficient to elucidate individual variation in parental investment and that other factors such as competition and risk of predation may also affect parental effort [50]–[52]. Indeed, food quality and predation risk seemed to interact to determine parental effort in orange-crowned warblers (*Oreothlypis celata*) where the rate at which parents fed their young was negatively correlated with nest predation risk [53]. Further, the potential effect of adult and brood predation may play a major role in shaping parental care levels not only via the effects on increased vigilance and mobbing efforts that may be traded-off against parental effort [40] but also due to the actual injury or predation of young and parents. That is because injury may reduce parental care levels. Furthermore, the predation of part of the brood or all of it will result in lower parental care levels or termination of a given breeding attempt, respectively. These events will force the parents or the left behind single parent to renest within the usually time-limited breeding season [51].

In conclusion, parent sunbirds adjusted their parental care levels according to the supplemented food that they consumed. As they consumed higher food quality, the parents spent more time flying to deliver more whole arthropods to their young. This suggests that the abundance and quality of food that only the parents consume can affect their ability to collect food for their young, not necessarily as a result of an increase in food abundance that is suitable for feeding only the nestlings.

Part of the parent sunbirds' ability to provide more food to their young was probably due to the time saved self-feeding while exploiting rich patches (e.g., the feeders). The addition of mealworms to a feeder probably allowed these parents to feed their nestlings more of the arthropods that they captured, representing the trade-off between parental self-feeding and feeding the young. The rest of the time savings and/or the increased total energy intake, from feeding on increasing food quality, were used differently by the sexes. The males increased their time spent mobbing near the nest and displaying in front of other females. Females, which usually have higher provisioning rates than males, used the extra time and/or energy to deliver even more food to their young. These increased differences in the levels of sex-specific roles suggest that each sex invests more in activities that it is already more prone to or maybe more efficient in performing when it has more time, energy and nutrients to do so.

## Acknowledgments

I thank Boaz Lederman for help in data collection and technical support. I further thank Berry Pinshow, Jon Wright, Arnon Lotem and Yoram Yom-Tov for valuable comments on an earlier version of this manuscript. I also thank Rainee Kaczorowski for valuable comments on the final version of this manuscript.
